# Establishment of a novel model of endometriosis-associated ovarian cancer by transplanting uterine tissue from Arid1a/Pten knockout mice

**DOI:** 10.1038/s41598-023-35292-4

**Published:** 2023-05-23

**Authors:** Motoki Ono, Tsutomu Miyamoto, Ryoichi Asaka, Junko Uchikawa, Hirofumi Ando, Yasuhiro Tanaka, Manaka Shinagawa, Yusuke Yokokawa, Shiho Asaka, Tian-Li Wang, Ie-Ming Shih, Tanri Shiozawa

**Affiliations:** 1 Department of Obstetrics and Gynecology, Shinshu University School of Medicine, 3-1-1 Asahi, Matsumoto, 390-8621 Japan; 2 Department of Laboratory Medicine, Shinshu University School of Medicine, 3-1-1 Asahi, Matsumoto, 390-8621 Japan; 3 Department of Diagnostic Pathology, Shinshu University Hospital, 3-1-1 Asahi, Matsumoto, 390-8621 Japan; 4 Department of Pathology, Johns Hopkins Medical Institutions, 1550 Orleans Street, CRB-2 Rm 306, Baltimore, MD 21287 USA; 5 Department of Gynecology and Obstetrics, Johns Hopkins Medical Institutions, 1550 Orleans Street, CRB-2 Rm 305, Baltimore, MD 21287 USA

**Keywords:** Biological techniques, Cancer, Molecular biology, Oncology, Pathogenesis

## Abstract

Although endometriosis is primarily benign, it has been identified as a risk factor for endometriosis-associated ovarian cancer (EAOC). Genetic alterations in *ARID1A*, *PTEN*, and *PIK3CA* have been reported in EAOC; however, an appropriate EAOC animal model has yet to be established. Therefore, the present study aimed to create an EAOC mouse model by transplanting uterine pieces from donor mice, in which *Arid1a* and/or *Pten* was conditionally knocked out (KO) in Pax8-expressing endometrial cells by the administration of doxycycline (DOX), onto the ovarian surface or peritoneum of recipient mice. Two weeks after transplantation, gene KO was induced by DOX and endometriotic lesions were thereafter removed. The induction of only *Arid1a* KO did not cause any histological changes in the endometriotic cysts of recipients. In contrast, the induction of only *Pten* KO evoked a stratified architecture and nuclear atypia in the epithelial lining of all endometriotic cysts, histologically corresponding to atypical endometriosis. The induction of *Arid1a*; *Pten* double-KO evoked papillary and cribriform structures with nuclear atypia in the lining of 42 and 50% of peritoneal and ovarian endometriotic cysts, respectively, which were histologically similar to EAOC. These results indicate that this mouse model is useful for investigating the mechanisms underlying the development of EAOC and the related microenvironment.

## Introduction

Endometriosis is characterized by the presence of endometrioid tissue outside of the uterus^1^. Ovaries and the pelvic peritoneum are common sites affected by endometriosis and form various lesions, such as endometriotic cysts and peritoneal endometriotic foci showing punctated, red, blue, brown, or white spots^2^. The pathogenesis of endometriosis is complex and several hypotheses, such as the implantation, metaplasia, and stem cell spread theories, have been proposed^1^. The “implantation theory” appears to be the most widely accepted among these theories. It posits that endometrial cells in menstrual blood flow back to the abdominal cavity through the fallopian tubes and implant at the ovary/peritoneum. Implanted tissue proliferates at the affected sites, and eventually forms endometriotic lesions^3^. In support of this hypothesis, previous studies demonstrated that eutopic and ectopic endometrial tissue shared the same somatic gene variants^4–6^.

Endometriosis is a common disease that affects approximately 10% of reproductive-aged women, with nearly 50% exhibiting dysmenorrhea, chronic pelvic pain, and infertility^7^. In recent years, ovarian endometriotic cysts (OEM) have been attracting attention as the origin of some types of ovarian cancer^3,8^. The histological types of endometriosis-associated ovarian cancer (EAOC) are primarily clear cell carcinoma and endometrioid carcinoma, and EAOC has been reported to develop in approximately 0.7% of OEM^8^.

The frequency of pathological variants in the AT-Rich Interaction Domain 1A (ARID1A), Phosphatase and Tensin Homolog (PTEN), and Phosphatidylinositol-4,5-Bisphosphate 3-Kinase Catalytic Subunit Alpha (PIK3CA) genes in EAOC tumors was found to be high, similar to endometrioid-type endometrial cancer (EEC)^9^. ARID1A is a crucial component of the SWI/SNF complex, which is involved in chromatin remodeling for the modulation of gene expression and is regarded as a tumor suppressor gene^10,11^. The loss of ARID1A function has been reported in various cancers, including 45% of EAOC and 40% of EEC^12–15^. PTEN and PIK3CA are both related to the PI3K/AKT/mTOR (PI3K) pathway, which is involved in various cellular functions associated with tumorigenesis, including proliferation, viability, and motility^16^. PIK3CA activates the PI3K pathway due to the synthesis of phosphatidylinositol-3,4,5-trisphosphate (PIP3). Therefore, the activated variants of PIK3CA are considered to be oncogenic^17^. On the other hand, PTEN functions as a negative regulator of the PI3K pathway by dephosphorylating PIP3 and acts as a tumor suppressor gene^18^. The loss of PTEN function has been reported in approximately 20% of EAOC and 70% of EEC^19,20^. Therefore, activation of the PI3K pathway and the loss of ARID1A function may be crucial for the development of EAOC and EEC.

Although EAOC is a neoplasm that has attracted the attention of many researchers, a number of difficulties are associated with its examination. The ectopic endometrioid epithelium lining OEM is often lost and replaced by granulation tissue^2^; therefore, it is challenging to obtain a sufficient number of OEM cells, the origin of EAOC, from clinical specimens. Primary cultures of OEM cells are also difficult. Alternatively, several animal models of benign endometriosis using rodents and baboons have been reported^21–23^. We only found one animal model of EAOC in the literature^24^; however, the reproducibility and feasibility of this model were insufficient. Our research group recently established a transgenic mouse that constantly and rapidly generates EEC by the doxycycline (DOX)-induced double knockout (KO) of the Arid1a and Pten genes^25^. Therefore, we herein attempted to create an EAOC mouse model by transplanting the above-described genetically engineered uterine tissue into donor mice.

## Results

### Establishment of peritoneal endometriosis in mice

**Figure 1:**
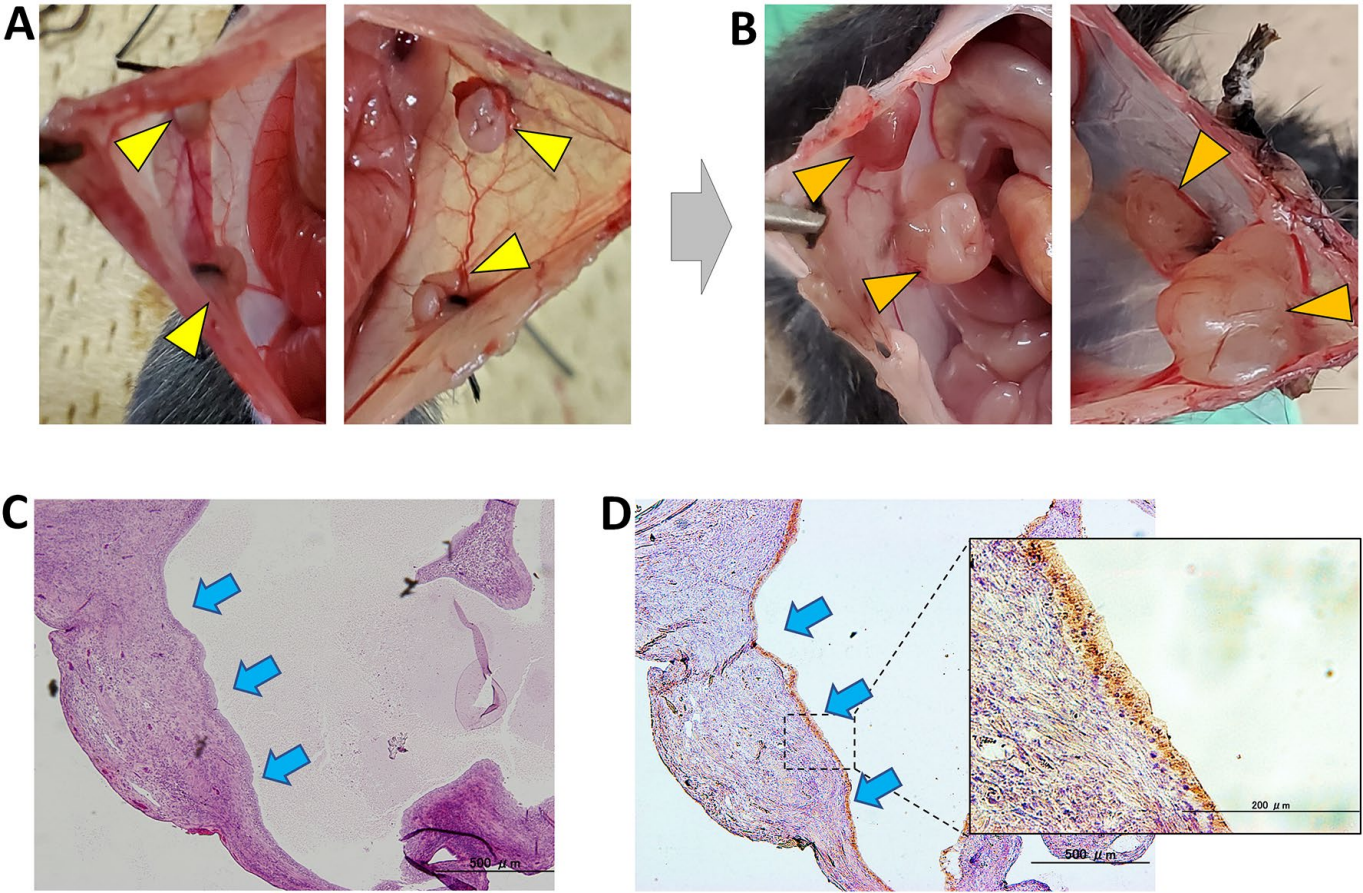
Ectopic endometrial tissue formation by the transplantation of small uterine pieces. (**A**) Small uterine fragments from a donor mouse were transplanted at four sites (yellow arrowheads) on the peritoneum of a recipient mouse. (**B**) Orange arrowheads indicate the cystic lesions that formed at the transplant sites 2 weeks after transplantation. (**C**,**D**) Microscopic photographs of the same cystic lesion by hematoxylin & eosin (HE) staining (**C**, 40) and Pax8 immunostaining (**D**, 40× and 200×). The cystic lesion is lined by a single layer of a Pax8-positive columnar epithelium (light blue arrow), indicating the formation of an ectopic endometrium (endometriosis).

We initially investigated whether transplanted uterine tissue from donor mice (wild-type C57BL/6) formed peritoneal endometriosis at the transplantation site in recipient mice of the same strain (Fig. 1A, Suppl. Fig. 1), as previously reported by Vernon et al.^21^. Recipient mice were euthanized 2 weeks after transplantation, and the intraperitoneal cavity was observed. The formation of cystic lesions was confirmed at the transplant sites in all mice (Fig. 1B). The fluid in cysts was transparent and slightly yellowish. Histopathological and immunohistochemical examinations revealed that a single layer of Pax8-positive columnar epithelial cells lined these cystic lesions (Fig. 1C,D). Since Pax8 is a specific marker of the Müllerian duct epithelium, including endometrial epithelial cells^26^, this result indicated that these Pax8-positive cells were derived from the endometrial epithelial cells of uterine pieces transplanted from the donor. Therefore, we considered the formation of ectopic endometrioid tissue or endometriosis using this method to be successful.

### Cancer induction in mouse peritoneal endometriotic cysts

**Figure 2:**
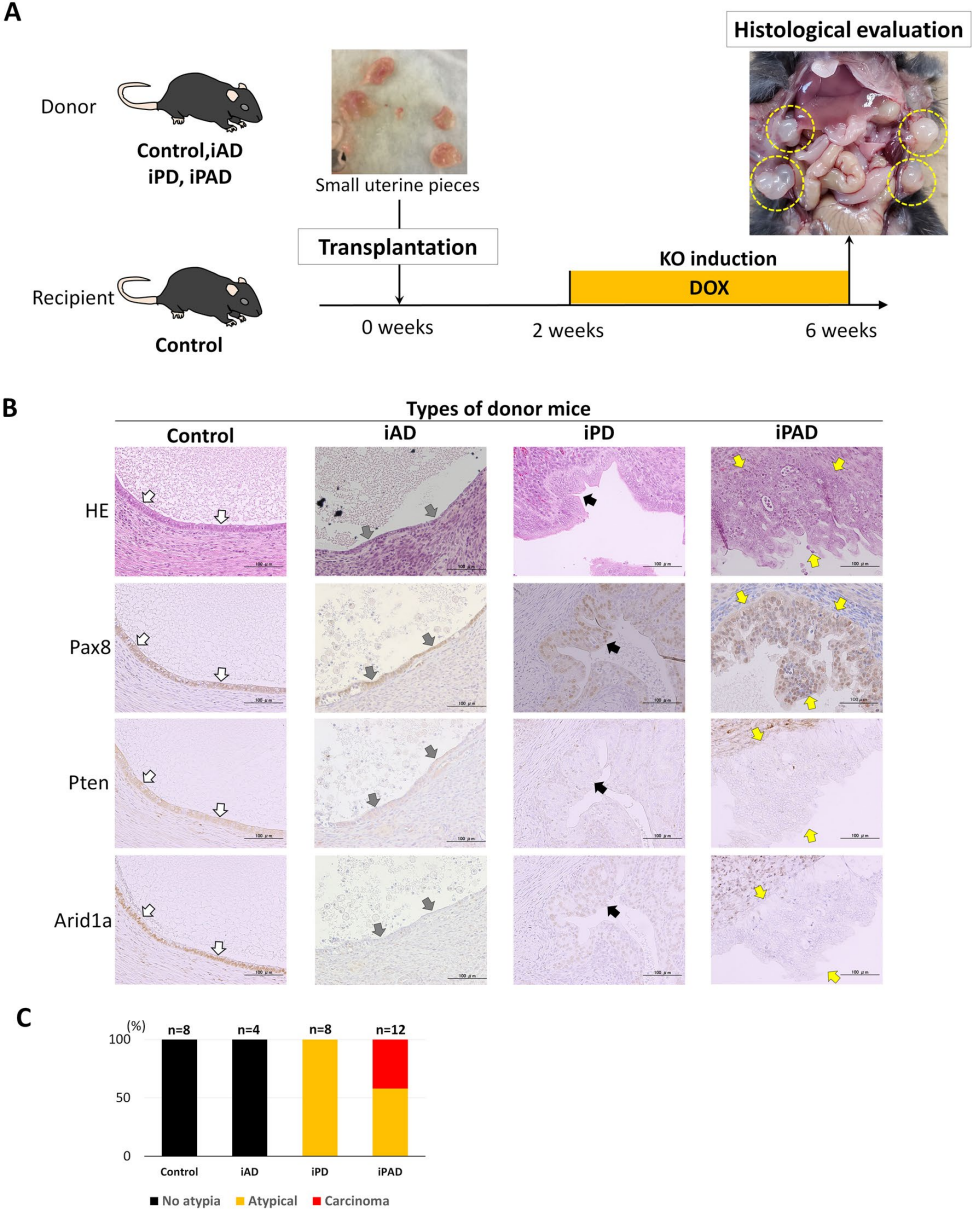
Induction of the knockout (KO) of Pten and Arid1a in peritoneal endometriosis. (**A**) Schema of the experiment. Yellow dashed circles indicate peritoneal cystic lesions that formed in this experiment. KO: knockout, DOX: oral administration of doxycycline to induce KO. (**B**) Microscopic photographs of the peritoneal cystic lesions from each donor type by hematoxylin & eosin (HE) staining and immunostaining for Pax8, Pten, and Arid1a (200×). Epithelial cells from all donor types are positive for Pax8. Cells from Control donor mice (white arrows) express both Pten and Arid1a. They show no morphological changes with single-layer growth without stratification and nuclear atypia. Cells from iAD donor mice (gray arrows) lack Arid1a expression, but show no morphological changes. Cells from iPD donor mice (black arrows) form focal lesions with stratification and mild to moderate nuclear atypia, corresponding to atypical endometriosis. In this lesion, cells lack the expression of Pten. Cells from iPAD donor mice (yellow arrows) form focal lesions with significant stratification, papillary growth, cribriform patterns, and prominent nuclear atypia corresponding to endometrioid carcinoma. In this lesion, cells lack the expression of both Pten and Arid1a. (**C**) The graph shows the ratio of lesion classifications formed by the knockout (KO) induction of Pten and Arid1a by the administration of doxycycline (DOX) in the peritoneal cystic lesions of each donor origin. Cysts from Control and iAD show no morphological changes (No atypia). All cysts (100%, 8/8) from iPD are classified as atypical endometriosis (Atypical). Approximately 50% of cysts (42%, 5/12) from iPAD are classified as endometrioid carcinoma (Carcinoma) and the others as atypical endometriosis (58%, 7/12).

We used control mice with wild-type *Pten* and *Arid1a* (*Pten*^WT/WT^, *Arid1a*^WT/WT^) as recipient mice. In control mice, Cre recombinase expression was induced in Pax8-positive cells by the oral administration of DOX; however, *Pten* and *Arid1a* cannot be KO by Cre because they are wild-type, not “floxed” genes flanked by loxP sequences. Three types of transgenic mice were used as donor mice: iAD (*Pten*^WT/WT^, *Arid1a*^flox/flox^), iPD (*Pten*^flox/flox^, *Arid1a*^WT/WT^), and iPAD (*Pten*^flox/flox^, *Arid1a*^flox/flox^), in which *Arid1a*-KO alone, *Pten*-KO alone, and the double-KO of *Pten* and *Arid1a*, respectively, were induced in Pax8-positive cells by the oral administration of DOX^25^. The administration of DOX was previously reported to produce orthotopic endometrial carcinoma within 10 days in 100% of iPAD mice and within 12 weeks in 50% of iPD mice, but not in iAD mice^25^. These transgenic mice were crossed with each other to ensure identical genetic backgrounds. DOX was administered to recipient mice 2 weeks after the implantation of small uterine pieces from the donors. Recipient mice were euthanized 4 weeks later and the intraperitoneal cavity was examined (Fig. 2A).

The formation of peritoneal cysts lined by Pax8-positive epithelial cells at the transplantation sites of all types of donor mice using these procedures was confirmed (Fig. 2). Figure 2B,C show the histological findings of the peritoneal endometriotic cysts of each donor mouse. In all 4 endometriotic cysts derived from two iAD mice, Pax8-positive epithelial cells did not express Arid1a and did not have architectural or nuclear atypia (Fig. 2B,C). In all 8 cysts derived from three iPD mice, Pax8-positive cells lacked the expression of Pten and showed stratification and nuclear atypia corresponding to atypical endometriosis (Fig. 2B,C). On the other hand, in 5 (42%) of the 12 cysts derived from three iPAD mice, cells on some parts of the Pax8-positive epithelium showed markedly stratified proliferation and abnormal structures, such as back-to-back and cribriform patterns, corresponding to endometrioid carcinoma (Fig. 2B). These lesions lacked the expression of both Pten and Arid1a. Therefore, we diagnosed these tumors as endometriosis-associated carcinomas. The remaining 7 cysts (58%) derived from iPAD mice were diagnosed as atypical endometriosis.

### Induction of EAOC in mice

We attempted to create the EAOC mouse model by transplanting small uterine pieces from genetically engineered mice onto the ovarian surface of control mice (Supplementary Fig. 1). In this experiment, we initially placed uterine tissues on the outside of the bursa, which covered the ovary; however, engraftment was not successful (data not shown). We then incised the ovarian bursa to expose the ovarian surface and sutured the serosal side of a small uterine graft directly onto the ovary. This approach resulted in the successful formation of OEM.

**Figure 3:**
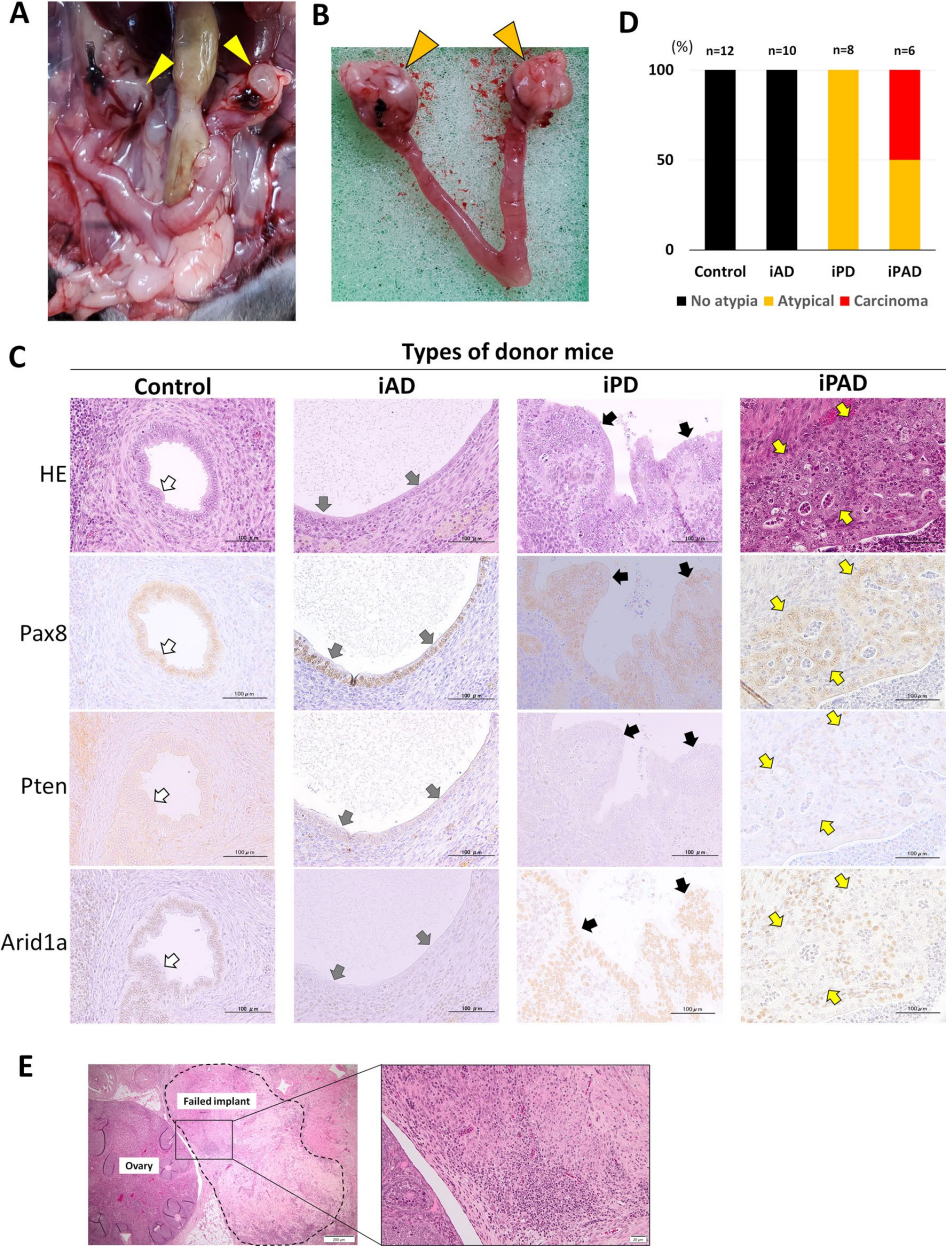
Induction of the knockout (KO) of Pten and Arid1a in ovarian endometriosis. (**A**) Laparotomy findings 6 weeks after the transplantation of small uterine pieces from the donor onto the ovaries and 4 weeks after the DOX treatment. Yellow arrowheads indicate bilateral ovaries. (**B**) The photo shows the removed uterus and bilateral ovaries (orange arrowheads). Both ovaries are enlarged. (**C**) Microscopic photographs show the cystic ovarian lesions from each donor type by hematoxylin & eosin (HE) staining and immunostaining for Pax8, Pten, and Arid1a (200×). Epithelial cells from all donor types are positive for Pax8. Cells from Control donor mice (white arrows) keep the expression of both Pten and Arid1a and show no morphological changes with single-layer growth. Cells from iAD donor mice (gray arrows) lack the expression of Arid1a, but show no morphological changes. Cells from iPD donor mice (black arrows) form focal lesions with stratified growth patterns and mild nuclear atypia, corresponding to atypical endometriosis. In this lesion, cells lack the expression of Pten. Cells from iPAD donor mice (yellow arrows) form focal lesions with back-to-back and cribriform growth patterns, stromal invasion, and prominent nuclear atypia, corresponding to endometrioid carcinoma. In this lesion, cells lack the expression of both Pten and Arid1a. (**D**) The graph shows the ratio of lesion classifications formed by the knockout (KO) induction of Pten and Arid1a by the administration of doxycycline (DOX) in the cystic ovarian lesions of each donor origin. Cysts from Control and iAD show no morphological changes (No atypia). All cysts (100%, 8/8) from iPD are classified as atypical endometriosis (Atypical). Approximately 50% of cysts (50%, 3/6) from iPAD are classified as endometrioid carcinoma (Carcinoma) and the others as atypical endometriosis (50%, 3/6). (**E**) This case’s implanted site from iPAD did not have a Pax8-positive epithelium (“Failed implant” area enclosed by a dashed line), suggesting engraftment failure.

The administration of DOX to recipient (control) mice was initiated 2 weeks after transplantation for wound healing, animals were euthanized 4 weeks later, and the ovaries and uterus were removed (Fig. 3A,B). A gross examination of the resected ovaries indicated that uterine tissue from donor mice transplanted onto the ovarian surface formed cystic structures with no clear borders from the original ovaries. Cystic epithelia were lined by a Pax8-positive epithelium, corresponding to OEM (Fig. 3B,C).

Twelve out of 12 and ten out of 14 ovaries transplanted with small pieces of the uterine tissues of Control and iAD, respectively, developed OEM with no structural or cellular atypia (Fig. 3C,D). The remaining four ovaries transplanted with iAD did not have a Pax8-positive ectopic endometrioid epithelium, suggesting engraftment failure. Eight out of 8 ovaries transplanted with the uterine tissue of iPD mice successfully developed OEM. The epithelia of OEM showed the cellular stratification of mild nuclear atypia, corresponding to atypical endometriosis (Fig. 3C,D). Six out of 8 ovaries developed OEM derived from iPAD donor mice, and the remaining two ovaries showed a Pax8-positive epithelium, suggesting engraftment failure (Fig. 3E). The overall engraftment rate with Pax8-positive epithelial cells was 86% (36/42 ovaries). In 3 out of the 6 OEM from iPAD mice, Pax8-positive cells in OEM partially lacked the expression of both Pten and Arid1a and showed cellular stratification, a cribriform structure, and stromal invasion with prominent nuclear atypia, corresponding to endometrioid carcinoma (Fig. 3C,D). Therefore, this tumor was diagnosed as EAOC. The remaining three OEM from iPAD mice were diagnosed as atypical endometriosis (Fig. 3D). Immunostaining confirmed that the administration of DOX did not reduce the expression of Pten or Arid1a in the eutopic endometrium of recipient mice (Supplementary Fig. 2).

## Discussion

By using the mouse endometriosis model described by Vernon et al.^21^, we herein successfully created a novel mouse EAOC model by transplanting small pieces of the uterine tissue of genetically engineered mice as the donor, in which *Pten* and/or *Arid1a* were KO in Pax8-positive endometrial cells^25^. This system conveniently enabled the development of EAOC in a relatively short period of time. Although several mouse models of ovarian cancer utilizing induced genetic mutations have been developed to date^11,27,28^, to the best of our knowledge, this is the first study to report a mouse EAOC model, which was further induced in the epithelia of OEM by the KO of relevant genes.

In this experiment, OEM was successfully formed by suturing the serosal side of a small uterine piece from a donor mouse directly onto the ovarian surface. Although a mouse model of peritoneal endometriosis induced by the surgical transplantation of small uterine pieces into the peritoneum or mesentery has already been established^21^, only a few studies have successfully generated OEM. Hayashi et al.^29^ recently reported new transplant techniques in which minced endometrial tissue pellets were directly attached to the ovarian surface after removing the ovarian bursa, and they successfully generated OEM. Wuyung et al.^24^ also generated OEM by suturing uterine tissues to the ovary; however, a description on the removal of the ovarian bursa was not provided. The unsuccessful formation of OEM without removal of the ovarian bursa suggests that direct contact between uterine tissue and the ovarian surface is critical for its formation.

The key to the success of our model is the avoidance of immune rejection. Therefore, matching of the genetic backgrounds of recipient mice (Control) and donor mice (iAD, iPD, and iPAD) is necessary. We herein backcrossed the mouse strains used in the experiment with C57BL/6 mice in three generations. In addition, each donor strain was crossbred with recipient mice to maintain a similar genetic background. As a result, many of the implanted uterine fragments were viable and successfully formed ectopic endometrioid tissue (endometriosis).

In the present mouse model, 100% of the uterine fragments transplanted into the peritoneum successfully engrafted and formed a Pax8-positive ectopic endometriotic epithelium, while some transplanted onto the ovarian surface did not. This result may be attributed to an insufficient blood supply because we did not perform revascularization of the grafts of small uterine pieces during transplantation surgery. In some recipient mice, only one of their ovaries had a Pax8-positive epithelium. This result supported the lack of this epithelium being due to a poor blood supply rather than rejection. In transplanted endometrioid epithelial cells derived from iAD, iPD, and iPAD mice, the oral administration of DOX partially induced the KO of Pten and Arid1a. Nevertheless, approximately 50% or more of epithelial cells lacked a response to DOX and retained the expression of Pten and Arid1a. This differed from previous findings showing the DOX-induced KO of Pten and Arid1a in all Pax8-positive epithelial cells on the orthotopic endometrium of these mouse strains^25^. In addition, the ratio of EAOC in the transplanted endometrioid epithelium derived from iPAD donor mice (50%) was lower than that of endometrial carcinoma in the eutopic endometria of the original iPAD mice (100%)^25^. The reason for these discrepancies between the transplanted and eutopic endometria of iPAD remains unclear; however, a poor blood supply in transplanted tissue may have reduced the accessibility of DOX to the transplanted ectopic endometrium. Another reason for the lower cancer ratio in recipient mice, into which the endometrium of iPAD mice was transplanted, may be related to the chemical characteristics of the cystic fluid of mouse OEM. Reactive oxygen species levels are high in the intra-cystic fluid of human OEM due to hemoglobin-related iron ions and inflammation-associated cytokines, and this unique microenvironment of OEM is considered to contribute to the development of EAOC^30,31^. However, in the present study, the intra-cystic fluid of OEM was clear and transparent, which differed from that in human OEM. Therefore, the lower incidence of cancer in the ectopic endometrioid tissue derived from iPAD donor mice may be due to a difference in the OEM microenvironment between the human and mouse models.

In the present study, EAOC developed as early as 4 weeks after the KO of *Pten* and *Arid1a*, but required more time to develop in other mouse models. Wilson et al. reported a unique mouse model in which endometrial cells with Arid1a and PIK3CA mutations, induced by an estrogen stimulation via Lactotransferrin-Cre, spread into the abdominal cavity by the uterotubal incision and salpingectomy, developing endometriosis at the ovaries or peritoneum^32^. A mouse model mimics the mechanism of developing endometriosis by the implantation of abnormal endometrial cells via retrograde menstruation. However, the ratio of cancer development has not been investigated. Cancers in the peritoneal cavity in Wilson’s mouse model are likely to be the peritoneal dissemination of endometrial cancer rather than endometriosis-associated cancers. Wuyung et al. auto-transplanted small uterine pieces into the ovary to form OEM in mice^24^. During transplantation, they sutured uterine fragments using threads coated with 7,12-dimethylbenz(a)anthracene, a well-known carcinogen. EAOC developed in 1 out of 5 mice (20%) at 10 weeks and in 5 out of 5 mice (100%) at 20 weeks. Chandler et al. reported a mouse model of ovarian clear cell-like carcinoma induced by *Arid1a*-KO and *Pik3ca* oncogenic variants via the ovarian injection of adenovirus-Cre^27^. Our method has an advantage over this method in that the induction of gene KO by the oral administration of DOX was more straightforward than the injection of adenovirus-Cre. Therefore, our EAOC mouse model will facilitate research on the various OEM microenvironments involved in EAOC carcinogenesis.

In conclusion, we successfully developed carcinoma by inducing the KO of Arid1a and Pten in the epithelium of endometriotic cysts, which were formed by the transplantation of small uterine pieces onto the peritoneum or ovarian surface. Although there are several issues that need to be resolved in order to induce cancer, such as the avoidance of rejection, this mouse model may help to elucidate the pathogenesis underlying the development of EAOC and facilitate the establishment of therapeutic methods.

## Methods

### Mouse strains including transgenic mice

C57BL/6 mice were purchased from The Jackson Laboratory Japan (Yokohama, Japan). All of the genetically engineered mouse strains used in the present study were previously established by and obtained from Johns Hopkins University^25^, and were kindly gifted to our laboratory at Shinshu University (see Supplementary Methods in Supplementary materials). The KO of *Pten* and/or *Arid1a* in Pax8-positive cells, including endometrial epithelial cells, may be induced in these transgenic mice by the administration of DOX through the Tet-on and Cre-loxP systems. Briefly, these mice have the reverse tetracycline transactivator (*rtTA*) gene after the promoter sequence of *Pax8*, a Müllerian duct epithelial marker including an endometrial epithelium, and the Cre recombinase (*Cre*) gene downstream of a tetracycline response element (*TRE*). Therefore, Pax8-positive cells express rtTA, which binds to TRE in the presence of DOX to induce the expression of Cre. Cre cuts and removes the target “floxed” gene region flanked by *loxP* sequences. Among transgenic mouse strains, the “control” has wild-type, not floxed *Pten* and *Arid1a* (*Pten*^WT/WT^, *Arid1a*^WT/WT^). “iAD (inducible *Arid1a* deletion)” has the *Arid1a* gene floxed in both alleles (*Pten*^WT/WT^, *Arid1a*^flox/flox^). “iPD (inducible *Pten* deletion)” has the *Pten* gene floxed in both alleles (*Pten*^flox/flox^, *Arid1a*^WT/WT^). “iPAD (inducible *Pten* and *Arid1a* deletion)” has the *Pten* and *Arid1a* genes floxed in both alleles (*Pten*^flox/flox^, *Arid1a*^flox/flox^).

### Creating peritoneal endometriosis

To create endometriotic lesions in the mouse abdominal cavity, we modified the method devised by Vernon and Wilson^21^. Ten-week-old donor mice were euthanized and their uteri were immediately removed. Small uterine pieces were prepared from the removed uteri using a 5-mm Derma punch^®^ (Maruho, Osaka, Japan) and surgically transplanted by silk suturing onto the peritoneum of 10-week-old recipient mice. Two weeks after transplantation, recipient mice were euthanized and their intraperitoneal lesions were observed and removed for a histological examination.

We performed all transplantation surgeries on recipient mice under appropriate anesthesia with isoflurane. We euthanized experimental mice by cervical dislocation under isoflurane anesthesia.

### Induction of Arid1a and Pten KO in peritoneal endometriosis

We used the 10-week-old transgenic mouse strains, including Control, iAD, iPD, and iPAD, as donors to create carcinoma in endometriotic cysts. Uterine pieces were transplanted into the peritoneum of recipient Control mice using the same method described above for the induction of peritoneal endometriosis. Two weeks after transplantation, we started the oral administration of 6 mg/body/day DOX, which was continued for 4 weeks to induce the KO of *Arid1a* and *Pten* in Pax8-positive cells derived from donors. Recipient mice were euthanized and their intraperitoneal lesions were removed for a histological examination.

### Induction of Arid1a and Pten KO in ovarian endometriosis

To create ovarian endometriosis, we transplanted small uterine pieces from donors onto the ovarian surface of recipients with silk suturing after removal of the ovarian bursa. We used 10-week-old Control, iAD, iPD, and iPAD mice as donors for uterine transplantation. Two weeks after transplantation, we started the oral administration of 6 mg/body/day DOX, which was continued for 4 weeks, to induce the KO of *Arid1a* and *Pten* in Pax8-positive cells derived from the donors. Recipient mice were euthanized, and their ovaries were removed for a histological examination.

### Pathological diagnosis and immunohistochemistry (IHC)

Removed tissues were processed to 4-µm-thick formalin-fixed and paraffin-embedded sections and utilized for hematoxylin and eosin staining or IHC for a histological diagnosis or the evaluation of KO, respectively. An ectopic endometrium (endometriosis) may be identified as Pax8-positive glandular epithelial cells in the cystic lesions that formed at the transplantation site of the donor’s uterine fragment. We diagnosed the histology of the mouse ectopic endometrium based on that of humans. Endometriosis without atypia was defined as a single layer of epithelial cells without stratification, a papillary structure, or nuclear atypia. On the other hand, epithelial cells in “atypical endometriosis” are characterized by various degrees of stratification, papillary changes, and nuclear atypia^33^. When ectopic endometrioid tissue shows a cribriform pattern or stromal invasion with prominent nuclear atypia, we diagnosed it as carcinoma.

We performed IHC as previously described^25,34^. Briefly, indirect immunostaining for Pax8, Arid1a, and Pten was conducted using an anti-Pax8 antibody (rabbit polyclonal, 1:1000 dilution, Thermo Fisher Scientific, Waltham, MA), anti-Arid1a antibody (rabbit polyclonal, 1:800 dilution, Sigma-Aldrich, Saint Louis, MO), and anti-Pten antibody (rabbit monoclonal, 1:100 dilution, Cell Signaling Technology, Danvers, MA), respectively, as primary antibodies. Tissue sections were deparaffinized in Hemo-De (FALMA, Tokyo, Japan) and rehydrated in a graded alcohol series. Antigens were retrieved by a microwave pretreatment in 0.01 M citrate buffer (pH 6.0) for 30 min. Sections were then treated with 0.3% hydrogen peroxide for 15 min to block endogenous peroxidase activity and incubated with the primary antibody at 4 °C overnight. After washing with phosphate-buffered saline, sections were incubated with a horseradish peroxidase-conjugated secondary antibody using Histofine MAX-PO (Nichirei, Tokyo, Japan) at room temperature for 60 min and stained with diaminobenzidine in 0.15% hydrogen peroxide. Counterstaining with hematoxylin was then performed.

### Ethics approval

In compliance with national regulations and guidelines, all mouse experiments were reviewed and approved by the Shinshu University Animal Experiment Committee (Approval No. 021094) and Shinshu University Genetic Recombination Experiment Committee (Approval No. 21-043) and were conducted in accordance with institutional guidelines and the Regulations for Shinshu University Animal Experimentation and Safety Management Regulations for Genetic Recombination Experiments at Shinshu University. The authors confirm that all animal experiments in the present study have been performed in accordance with ARRIVE guidelines.

## Supplementary Information

Supplementary Information.

## Data Availability

All data generated or analyzed during this study are included in this published article and its supplementary information files.
