# Supplementary material for: Establishment of a novel model of endometriosis-associated ovarian cancer by transplanting uterine tissue from Arid1a/Pten knockout mice
Source: Sci Rep. 2023 May 23;13:8348. doi: 10.1038/s41598-023-35292-4 (PMC10205720; doi:10.1038/s41598-023-35292-4)
Supplement: Supplementary file 1 — Supplementary Information. [file 41598_2023_35292_MOESM1_ESM.pdf]

Supplementary materials

Supplementary Figure 1: Transplantation procedure

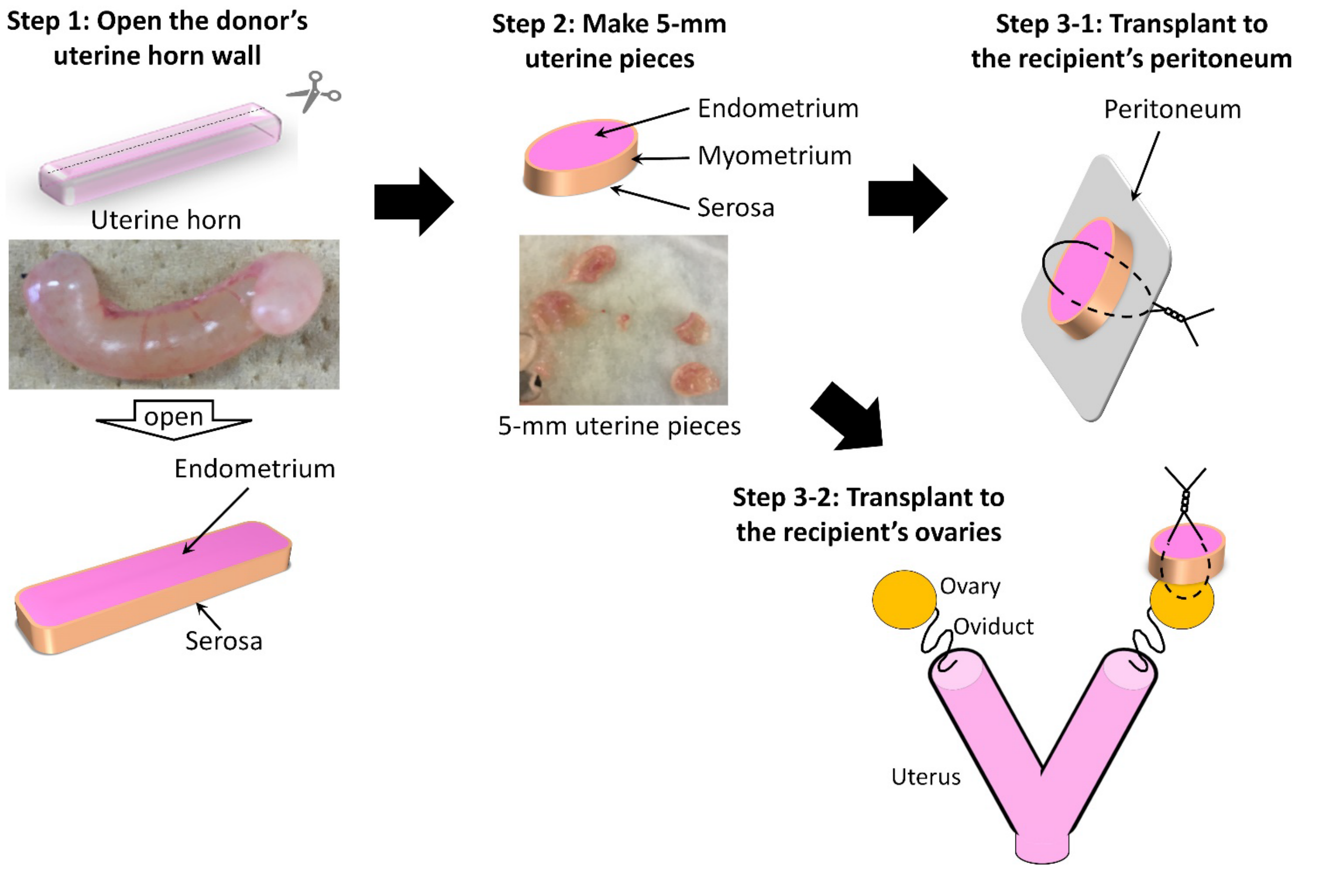

Step 1: Open the uterine horn wall of the donor through a longitudinal incision immediately after removal.

Step 2: Make 5-mm uterine pieces from the whole layer of the uterine wall, including the endometrium and serosa.

Step 3: Transplant small uterine pieces by directly attaching the serosal site to the peritoneum (**Step 3-1**) or ovarian surface (**Step 3-2**) of the recipient.

Supplementary Figure 2

Immunohistochemistry of the eutopic endometrium in the recipient mouse after DOX administration

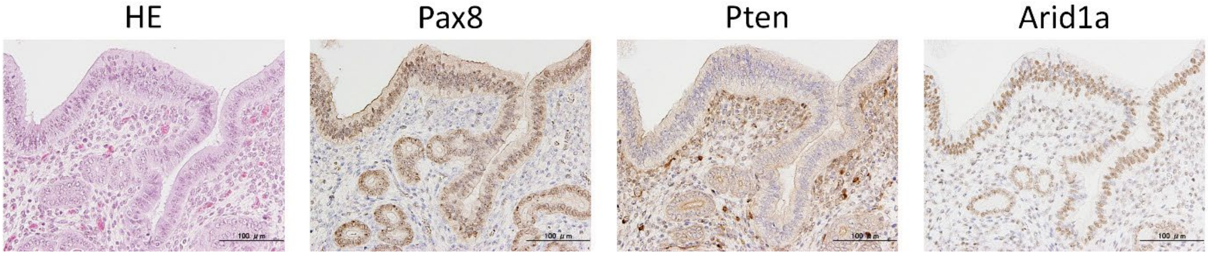

Photos are hematoxylin and eosin (HE) and immunohistochemical staining for Pax8, Pten, and Arid1a in the eutopic endometrium of a recipient mouse (Control: *Pten*$^{WT/WT}$, *Arid1a*$^{WT/WT}$) transplanted with a small uterine fragment onto the peritoneum from a iPAD mouse (*Pten*$^{flox/flox}$, *Arid1a*$^{flox/flox}$) and administered doxycycline (DOX). These photos demonstrated that Pax8-positive eutopic endometrial epithelial cells in the recipient mouse retained the expression of Pten and Arid1a.

Supplementary Methods

Mouse strains and genotyping

The *Arid1a*$^{\text{flox/flox}}$ mouse strain originating from 129P2/OlaHsd was previously described [1]. This strain has exon 8 of the *Arid1a* gene flanked by *loxP* sequences. Cre removes *Arid1a* exon 8 and introduces a frameshift mutation and premature stop codon (p.Gly809Hisfs*6) [2]. The genotype was confirmed by PCR using primers (Forward GTAATGGGAAAGCGACTACTGGAG, Reverse TGTTCATTTTTGTGGCGGGAG). The product size of wild-type (WT), floxed, and recombined Arid1a by Cre were 669, 845, and 298 bp, respectively.

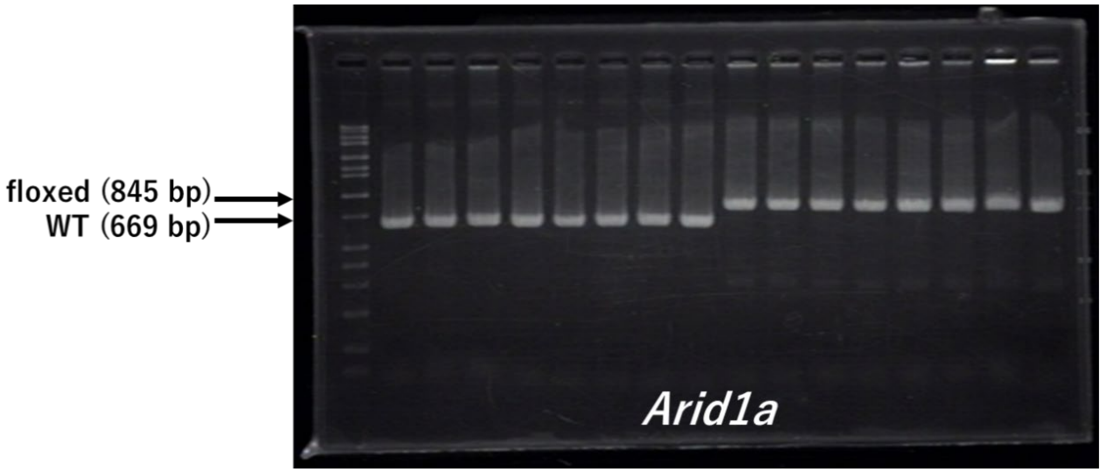

The *Pten*$^{\text{flox/flox}}$ mouse strain was B6.129S4-*Pten*$^{\text{tm1Hwu}}$/J originally from the Jackson Laboratory [3]. This strain possesses *loxP* sites flanking exon 5 of the *Pten* gene. The genotype was confirmed by PCR using primers (Forward CAAGCACTCTGCGAACTGAG, Reverse AAGTTTTTGAAGGCAAGATGC). The product sizes of wild-type (WT) and floxed *Pten* were 150 and 300 bp, respectively.

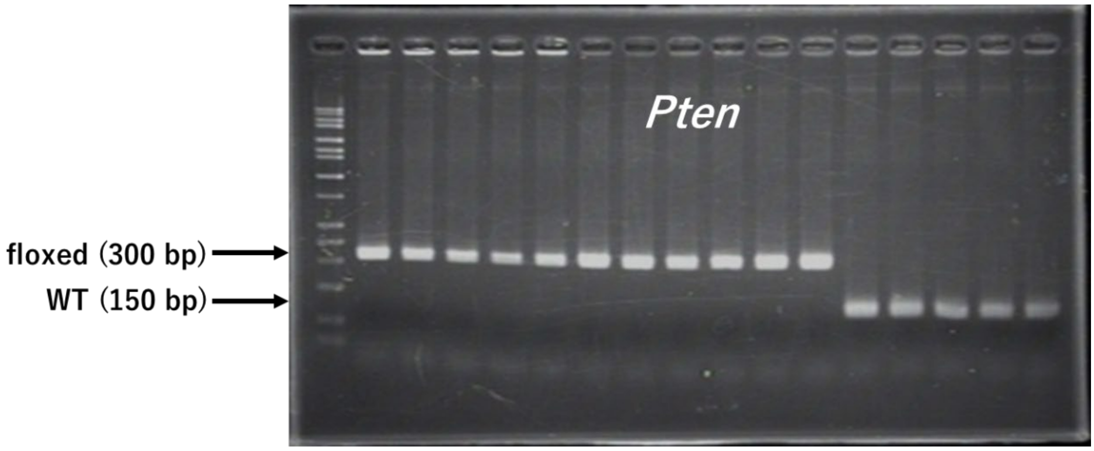

The *Pax8-rtTA* mouse strain was Tg(*Pax8-rtTA*2S*M2)1Koes/J from the Jackson Laboratory [4]. The genotype was confirmed by PCR using primers (Forward CCATGTCTAGACTGGACAAGA, Reverse CTCCAGGCCACATATGATTAG). The product size of *Pax8-rtTA* was 650 bp. The *TRE-Cre* (*tetO-Cre*) mouse strain was also from the Jackson Laboratory [4]. The genotype was confirmed by PCR using primers (Forward GCGGTCTGGCAGTAAAAACTATC, Reverse GTGAAACAGCATTGCTGTCACTT). The product size of *TRE-Cre* was 100 bp.

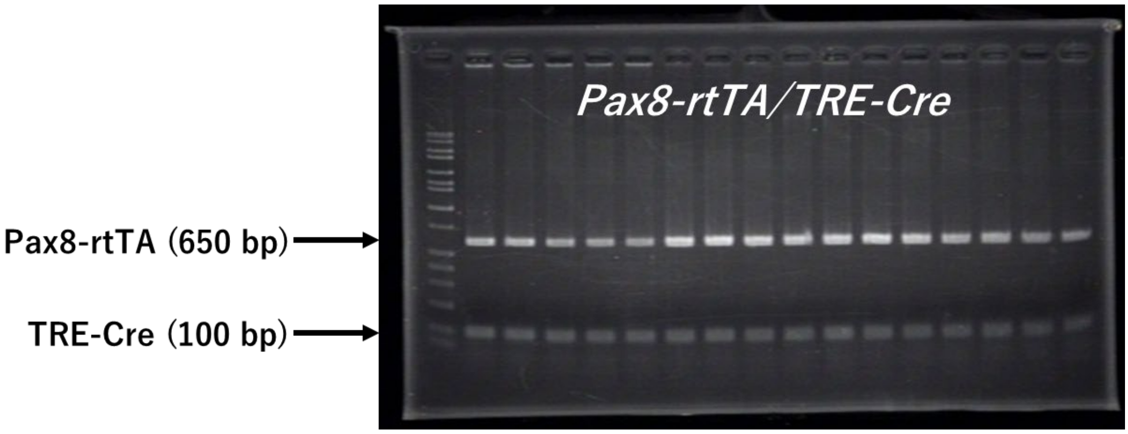

Mice bearing *Pax8-rtTA*, *TRE-Cre*, floxed *Pten*, and floxed *Arid1a* alleles were previously generated by cross-breeding the above transgenic mouse strains [5]. Two male and two female mice carrying these four alleles were kindly gifted from Johns Hopkins University and maintained in our laboratory at Shinshu University. These mice were bred on a mixed background of C57BL/6, DBA, and S129 and had already been backcrossed to C57BL/6 several times at Johns Hopkins University.

Male mice bearing all four alleles were backcrossed three times with C57BL/6J females at our laboratory. Progeny positive for appropriate alleles were intercrossed to generate the following four strains:

- Control: *Pax8-rtTA*, *TRE-Cre*, $Pten^{WT/WT}$, $Arid1a^{WT/WT}$
- iAD (inducible *Arid1a* deletion): *Pax8-rtTA*, *TRE-Cre*, $\mathrm{Pten}^{WT/WT}$, $Arid1a^{flox/flox}$
- iPD (inducible *Pten* deletion): *Pax8-rtTA*, *TRE-Cre*, $Pten^{flox/flox}$, $Arid1a^{WT/WT}$
- iPAD (inducible *Pten* and *Arid1a* deletion): *Pax8-rtTA*, *TRE-Cre*, $Pten^{flox/flox}$, $Arid1a^{flox/flox}$

Female control mice were used as the “recipient” in the present study.

[1] Gao X, Tate P, Hu P, and Wang Z. ES cell pluripotency and germ-layer formation require the SWI/SNF

chromatin remodeling component BAF250a. *Proc Natl Acad Sci U S A.* **105**, 6656-61 (2008)

[2] Guan B, et al. Roles of Deletion of Arid1a, a Tumor Suppressor, in Mouse Ovarian Tumorigenesis. *JNCI.* **106**, dju146 (2014).

[3] Groszer M, et al. Negative regulation of neural stem/progenitor cell proliferation by the *Pten* tumor suppressor gene in vivo. *Science.* **294**, 2186-2189 (2001)

[4] Perets, R. et al. Transformation of the fallopian tube secretory epithelium leads to high-grade serous ovarian cancer in *Brca*;*Tp53*;*Pten* models. *Cancer Cell* **24**,751–765 (2013).

[5] Rahmanto YS, et al. Inactivation of Arid1a in the endometrium is associated with endometrioid tumorigenesis through transcriptional reprogramming. *Nat. Commun*. **11**, 1–14 (2020).
